# Catalytic Asymmetric Nitro-Mannich Reactions with a Yb/K Heterobimetallic Catalyst

**DOI:** 10.3390/molecules15031280

**Published:** 2010-03-04

**Authors:** Tatsuya Nitabaru, Naoya Kumagai, Masakatsu Shibasaki

**Affiliations:** Graduate School of Pharmaceutical Sciences, The University of Tokyo, 7-3-1 Hongo, Bunkyo-ku, Tokyo 113-0033, Japan; E-Mail: ff097020@mail.ecc.u-tokyo.ac.jp (T.N.)

**Keywords:** nitro-Mannich, ytterbium, heterobimetallic, asymmetric catalysis, amide-based ligand

## Abstract

A catalytic asymmetric nitro-Mannich (aza-Henry) reaction with rare earth metal/alkali metal heterobimetallic catalysts is described. A Yb/K heterobimetallic catalyst assembled by an amide-based ligand promoted the asymmetric nitro-Mannich reaction to afford enantioenriched *anti*-β-nitroamines in up to 86% ee. Facile reduction of the nitro functionality allowed for efficient access to optically active 1,2-diamines.

## 1. Introduction

**Scheme 1:**
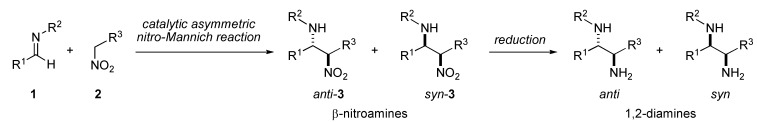
Catalytic asymmetric nitro-Mannich reaction for the synthesis of 1,2-diamines.

The catalytic asymmetric nitro-Mannich (aza-Henry) reaction is a useful carbon-carbon bond-forming reaction that assembles imines **1** and nitroalkanes **2** under proton-transfer conditions, affording enantiomerically enriched β-nitroamines **3** [1,2]. Facile reduction of the nitro functionality of the nitro-Mannich product to amines allowed for efficient access to synthetically versatile optically active 1,2-diamines (Scheme 1) [3,4,5]. Due to its synthetic utility, increasing efforts have been directed toward developing an efficient catalytic asymmetric nitro-Mannich reaction. Since our report on the catalytic asymmetric nitro-Mannich reaction with a binaphthol-based heterobimetallic catalyst [6], various metal-based catalysts [7,8,9,10,11,12,13] and organocatalysts [14,15,16,17,18,19,20,21,22,23,24,25] have been uncovered for diastereo- and enantioselective nitro-Mannich reactions. We previously developed a highly *anti-* and enantioselective nitroaldol reaction with a rare earth metal/alkali metal heterobimetallic catalytic system assembled by an amide-based chiral ligand [26,27]. In this catalyst design, bifunctional catalysis [28,29,30,31,32] exerted by an Nd/Na bimetallic catalyst, where the Nd cation acts as a Lewis acid and the Na–aryloxide acts as Brønsted base, is key to achieving high catalytic activity and stereoselectivity. In our continuing program to expand the utility of the heterobimetallic bifunctional catalyst organized by the amide-based ligand [33,34,35,36,37], we planned to develop a diastereo- and enantioselective nitro-Mannich reaction based on rare earth metal/alkali metal heterobimetallic catalysis.

## 2. Results and Discussion

### 2.1. Identification of a Suitable Heterobimetallic Catalyst for Asymmetric Nitro-Mannich Reaction

**Scheme 2:**
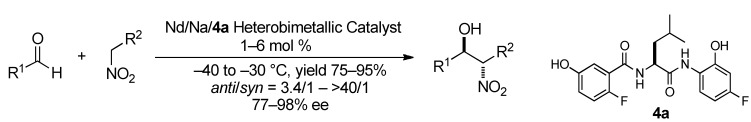
Catalytic asymmetric nitroaldol reaction promoted by Nd/Na/**4a** heterobimetallic catalyst.

Initial trials were performed to identify the best combination of rare earth metals and alkali metals with an amide-based ligand **4a**, which is the best ligand for a catalytic asymmetric nitroaldol reaction of aldehydes and nitroalkanes [27]. In the model reaction of Boc-imine **1a** [38] derived from benzaldehyde and nitroethane (**2a**), various heterobimetallic catalysts were prepared from rare earth metal alkoxides [RE(O*^i^*Pr)_3_] and alkali metal sources and evaluated based on the chemical yield and stereoselectivity of the desired product **3aa** (Table 1). The Nd/Na/**4a** heterogeneous heterobimetallic catalyst, which was isolated as an insoluble material in THF, afforded high stereoselectivity in *anti*-selective asymmetric nitroaldol reactions (Scheme 2), but gave poor results in the asymmetric nitro-Mannich reaction, affording *anti*-**3aa** preferentially in 87% yield with *anti*/*syn* = 3.7/1 and 0% ee (*anti*) (entry 1). Assuming that aldehyde and imine **1a** have different coordination modes, it is reasonable that the Nd/Na/**4a** catalytic system failed to exhibit highly stereoselectivity. The use of other alkali metals led to a substantial loss in catalytic activity (entries 2,3), therefore we searched for other rare earth metals. The heterobimetallic catalytic system comprising various rare earth metals and Na exhibited generally high catalytic activity to afford **3aa** in high yield with moderate *anti*-selectivity by using 3 mol % of catalyst loading, whereas the dominant *anti*-diastereomer was nearly racemic (entries 4–8). Thus, we turned our attention to the use of other alkali metals. Despite the low catalytic activity of RE/Li/**4a** catalysts, RE/K/**4a** catalysts promoted the desired reaction (Table 2). Although the low catalytic activity was observed for catalysts prepared from KHMDS compared with their NaHMDS counterparts, promising *anti*-selectivity and enantioselectivity were obtained, particularly when using Er or Yb as rare earth metals (entries 6,7). The low catalytic activity was compensated for by using 10 mol % of catalyst. Decreasing the catalyst loading to 5 mol % resulted in a marginal loss of enantioselectivity (entry 8).

**Table 1:** Catalytic asymmetric nitro-Mannich reaction promoted by RE/alkali metal/**4a** heterobimetallic catalyst.*^a^*

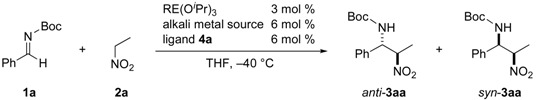
**Entry**	**RE(O *^i^* Pr)_3_**	**Alkali metal source *^d^***		**Time (h)**	**Yield *^e^* (%)**	**dr (*anti*/*syn*)**	**ee (*anti*) (%)**
1 *^b^*	Nd_5_O(O *^i^*Pr)_13_*^c^*	NaHMDS		21	87	3.7/1	0
2	Nd_5_O(O *^i^*Pr)_13_*^c^*	LHMDS		21	trace	ND	ND
3	Nd_5_O(O *^i^*Pr)_13_*^c^*	KHMDS		21	trace	ND	ND
4	La(O *^i^*Pr)_3_	NaHMDS		19	72	7.2/1	7
5	Sm(O *^i^*Pr)_3_	NaHMDS		19	89	3.5/1	11
6	Gd(O *^i^*Pr)_3_	NaHMDS		19	80	4.4/1	8
7	Er(O *^i^*Pr)_3_	NaHMDS		19	83	5/1	3
8	Yb(O *^i^*Pr)_3_	NaHMDS		19	92	5.2/1	6

*^a^* **1a**: 0.3 mmol, **2a**: 3.0 mmol. *^b^* A heterogeneous complex formed during catalyst preparation procedure was isolated by centrifugation and used as catalyst. *^c^* oxo-Complex of Nd(O*^i^*Pr)_3_. The amount used was calculated based on Nd. *^d^* HMDS: hexamethyldisilazane. *^e^* Determined by ^1^H-NMR analysis with Bn_2_O as an internal standard.

**Table 2:** Catalytic asymmetric nitro-Mannich reaction promoted by RE/K/**4a** heterobimetallic catalyst.*^a^*

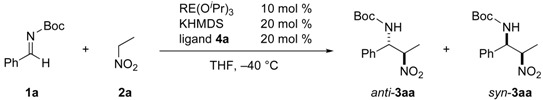
**Entry**	**RE(O*^i^*Pr)_3_**		**Time (h)**	**Yield*^b^*(%)**	**dr (*anti*/*syn*)**	**ee (*anti*) (%)**	
1	La(O*^i^*Pr)_3_		27	0	ND	ND	
2	Pr(O*^i^*Pr)_3_		27	66	5.3/1	6	
3	Sm(O*^i^*Pr)_3_		27	73	5.8/1	18	
4	Gd(O*^i^*Pr)_3_		27	66	7.7/1	0	
5		Dy(O*^i^*Pr)_3_	17	61	6.7/1	20	
6		Er(O*^i^*Pr)_3_	17	68	9.0/1	51	
7		Yb(O*^i^*Pr)_3_	27	72	11/1	68	
8		Yb(O*^i^*Pr)_3_	17	78	6.6/1	55	

*^a^* **1a**: 0.1 mmol, **2a**: 1.0 mmol. *^b^*Determined by ^1^H-NMR analysis with Bn_2_O as an internal standard.

**Figure 1:**
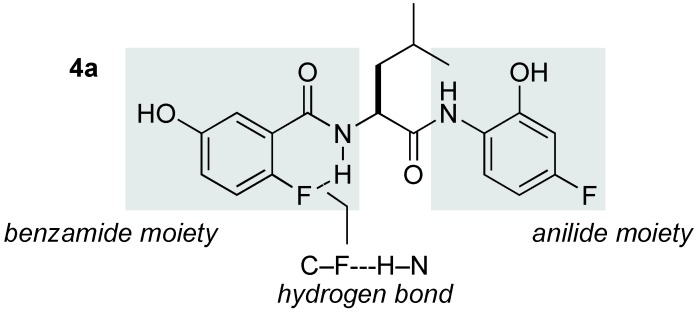
Intramolecular hydrogen bond in ligand **4a**.

We next focused on the effect of the amide-ligand architecture on stereoselectivity. **4a** was developed in the study of the asymmetric nitroaldol reaction, and its 2-fluoro substituent on the benzamide moiety was crucial for limiting the C–C bond rotation through the C–F---H–N hydrogen bond (Figure 1) [39,40]. The nitro-Mannich reaction with a Yb/K catalytic system prepared from various amide-based ligands is summarized in Table 3. A ligand lacking 2-fluoro substituent **4b** and a pyridine-type ligand **4c** gave almost racemic product, suggesting that conformational restriction of the 3-hydroxybenzamide moiety was a significant factor (entries 2,3). We then examined the effect of modifying the anilide moiety by ligands **4d** and **4e**. Interestingly, both the presence and the position of the fluoro substituent affected stereoselectivity, and the catalyst derived from **4e** predominantly afforded the opposite enantiomer, presumably because the pattern of the oligomeric association of the heterobimetallic catalyst was different (entries 4,5). Together, these data indicated that the catalyst comprising Yb/K/**4a** was optimum for the present reaction; therefore, the final optimization of reaction conditions was conducted on a Yb/K heterobimetallic system (Table 4). The ratio of Yb/K/**4a** was a dominant factor for both catalytic activity and stereoselectivity, revealing that Yb/K/**4a** = 1/2/2 was optimal for catalytic performance (entries 1–4). A significant solvent effect was observed, likely because the association of the ligand through hydrogen bonding and metal coordination was susceptible to coordinative characteristics and/or the dielectric constant of the solvents (entries 5–8) [41]. Non-polar, non-coordinating solvents exhibited poor catalytic efficiency (entries 5,6). Lowering the reaction temperature to –60 °C somewhat increased both diastereo- and enantioselectivity, affording **3aa** in *anti*/*syn* = 18/1 and 73% ee (*anti*) (entry 9).

**Table 3:** Catalytic asymmetric nitro-Mannich reaction promoted by RE/K/**4** heterobimetallic catalysts.

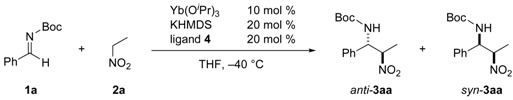
**Entry**	**Amide-based ligand 4**		**Time (h)**	**Yield *^c^* (%)**	**dr (*anti*/*syn*)**	**ee (*anti*) (%)**
1	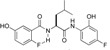	**4a**	27	72	11/1	68
2	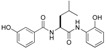	**4b**	23	88	3.1/1	17
3	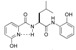	**4c**	17	91	2.9/1	2
4	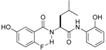	**4d**	23	68	4.5/1	19
5	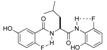	**4e**	23	66	4.5/1	31 *^b^*

*^a^***1a**: 0.1 mmol, **2a**: 1.0 mmol. *^b^*The opposite enantiomer was obtained preferentially. *^c^*Determined by ^1^H-NMR analysis with Bn_2_O as an internal standard.

**Table 4:** Optimization of reaction conditions based on Yb/K/**4a** heterobimetallic catalyst.*^a^*

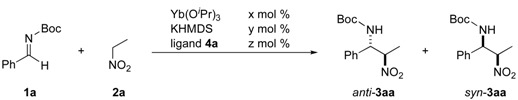
**Entry**	**x**	**y**	**z**	**Solvent**	**Temp (°C)**	**Time (h)**	**Yield *^b^* (%)**	**dr (*anti*/*syn*)**	**ee (*anti*) (%)**
1	10	20	20	THF	–40	27	72	11/1	68
2	10	10	10	THF	–40	13	23	3.8/1	17
3	10	20	10	THF	–40	13	72	2.3/1	4
4	10	10	20	THF	–40	13	62	7.6/1	52
5	10	20	20	CH_2_Cl_2_	–40	12	26	4.4/1	4
6	10	20	20	toluene	–40	12	46	2.2/1	4
7	10	20	20	EtOAc	–40	12	79	3.1	20
8	10	20	20	*^t^*BuOMe	–40	12	70	7.6	52
9	10	20	20	THF	–60	24	77	18/1	73

*^a^***1a**: 0.1 mmol, **2a**: 1.0 mmol. *^b^*Determined by ^1^H NMR analysis with Bn_2_O as an internal standard.

### 2.2. Scope of the Catalytic Asymmetric Nitro-Mannich Reaction with Yb/K/4a Heterobimetallic Catalyst

**Scheme 3:**
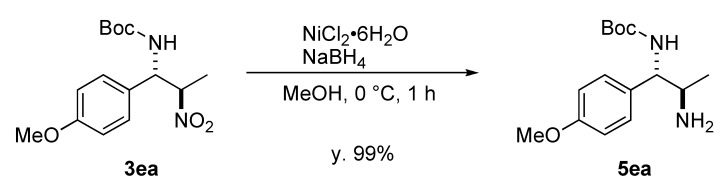
Reduction of nitro group of the niro-Mannich product.

With a heterobimetallic Yb/K/**4a** catalyst for the nitro-Mannich reaction in hand, we examined the substrate generality of the catalytic system (Table 5). Imines bearing a 2-naphthyl or tolyl group gave the corresponding nitro-Mannich products **3ba**–**3da** with stereoselectivity comparable to **3aa** (entries 2–4), indicating that steric issue was not significant in stereoselectivity. On the other hand, the stereoselectivity appeared to be dependent on the electronic nature of the aromatic group of imines **1**; an imine with electron-withdrawing substituents afforded the product in poor stereoselectivity whereas an imine with an electron-donating substituent enhanced stereoselectivity (entries 5–7), suggesting that a background racemic pathway was involved [42]. Diastereo- and enantioselectivity were uniform in the course of the reaction, indicating that the possibility of retro-reaction and epimerization of the product were neglected [43]. The nitro group of the nitro-Mannich product *anti*-**3ea** was readily reduced to generate *anti*-1,2-diamine **5ea** (Scheme 3), indicating the synthetic utility of the nitro-Mannich reactions to access synthetically versatile enantioenriched 1,2-diamines.

**Table 5:** Substrate scope of nitro-Mannich reaction promoted by Yb/K/**4a** heterobimetallic catalyst.*^a^*

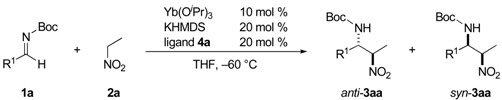
**Entry**	**R^1^**		**Product**		**Time (h)**	**Yield *^b^* (%)**	**dr (*anti*/*syn*)**	**ee (*anti*) (%)**
1	Ph	**1a**	**3aa**		22	80	18/1	73
2	2-naph	**1b**	**3ba**		22	71	17/1	72
3	4-Me	**1c**	**3ca**		44	87	22/1	86
4	3-Me	**1d**	**3da**		20	76	13/1	75
5	4-OMe	**1e**	**3ea**		44	79	19/1	82
6	4-Cl	**1f**	**3fa**		20	74	6.6/1	50
7	4-CF_3_	**1g**	**3ga**		20	72	2.4/1	14

*^a^* **1a**: 0.3 mmol, **2a**: 3.0 mmol. *^b^* Isolated yield.

## 3. Experimental

### 3.1. General

Reactions were performed in flame-dried 20 mL test tubes with a magnetic stirring bar unless otherwise noted. The test tubes were fitted with a glass 3-way stopcock and reactions were conducted under argon atmosphere. Air- and moisture-sensitive liquids were transferred via gas-tight syringe and stainless-steel needle. Flash chromatography was performed using silica gel 60 (230-400 mesh) purchased from Merck. Commercial reagents were purchased from Kojundo Chemical Co. Ltd. (RE(O*^i^*Pr)_3_, RE_5_O(O*^i^*Pr)_13_: Stored and handled in a dry box, contact: http://www.kojundo.co.jp/English/index.html, Fax: +81-49-284-1351, E-Mail: sales@kojundo.co.jp.), TCI (aldehydes, nitroethane, nitropropane), and Aldrich (LHMDS (1.0 M/THF), NaHMDS (1.0 M/THF), and KHMDS (0.5 M/toluene)). *N*-Boc imines **1** were prepared by following reported procedure [38]. THF was distilled from sodium/benzophenone ketyl. Other dry solvents were used as received from KANTO Chemical Co. Ltd. ^1^H, ^13^C NMR spectra were recorded on JEOL LA-500 or ECX–500 spectrometers (500 MHz). Chemical shifts for protons are reported in parts per million downfield from tetramethylsilane and are referenced to residual protium in the NMR solvent (CDCl_3_: δ 7.24 ppm). Chemical shifts for carbons are reported in parts per million downfield from tetramethylsilane and are referenced to the carbon resonances of the solvent (CDCl_3_: δ 77.0). Coupling constants are reported in Hertz (Hz). Infrared (IR) spectra were obtained using a JASCO FT/IR 410 spectrophotometer. ESI mass spectral data for new compounds were obtained using a JEOL AccuTOF JMS-T100LC mass spectrometer. Melting point was recorded on Yamato Melting Point Apparatus Model MP-21. All the nitro-Mannich products were reported in the literature and spectral data of them were matched to the reported ones.

### 3.2. General Procedure for Catalytic Asymmetric Nitro-Mannich Reaction with Yb/K/***4a*** Heterobimetallic Catalyst (Table 5, Entry 1)

To a flame dried 20 mL test tube charged with ligand **4a** (22.7 mg, 0.06 mmol) and dried under vacuum at room temperature for 10 min. Ar was back-filled to the test tube, then THF (520 μL), Yb(O*^i^*Pr)_3_ (341 μL, 0.03 mmol, 0.0878 M/THF), and KHMDS (120 μL, 0.06 mmol, 0.5 M/toluene) were added by well-dried syringe and needle successively at room temperature, leading to a white suspension. The subsequent addition of nitroethane (**2a**, 215 μL, 3.0 mmol) at the same temperature gave a clear catalyst solution. The test-tube was immersed to an electronically-controlled cooling bath at −60 °C, then THF solution (300 μL) of *N*-Boc-imine **1a** (61.5 μL, 0.30 mmol) was added to run the reaction. After stirring the reaction mixture at the same temperature for 22 h, 1N HCl aq. was added and the resulting mixture was extracted with ethyl acetate (×2). The combined organic layers were washed with sat. aq. NaHCO_3_ and brine, then dried over Na_2_SO_4_. After the removal of volatiles under reduced pressure, the resulting residue was analyzed by ^1^H NMR to determine diastereomeric ratio (*anti*/*syn* = 18/1) of **3aa** (PhC***H***(NHBoc)-: *anti* δ 5.18 ppm;*syn* δ 5.09 ppm). The residue was purified by silica gel column chromatography (*n*-hexane/ethyl acetate = 20/1 to 4/1) to give **3aa** as a coloress solid (67.5 mg, 0.241 mmol, 80% yield). Spectroscopic data of the obtained **3aa** were matched to the reported data (reg# 1001022-93-0). Enantiomeric excess was determined by HPLC analysis (*anti* = 73% ee, DAICEL CHIRALPAK AD-H (*φ* 0.46 cm x 25 cm), 2-propanol/*n*-hexane 1/9, flow rate 1.0 mL/min, detection at 210 nm, t_R_ 8.0 min [*anti* minor-enantiomer: (*1S*,*2R*)] and 8.8 min [*anti* major-enantiomer: (*1R*,*2S*)]. Absolute configuration was determined by comparing the reported retention time in HPLC analysis of the stereochemically defined sample [18]. The nitro-Mannich products **3ba**–**3fa** are previously reported compounds. **3ba**: CAS 1001023-00-2, **3ca**: CAS 1001022-97-4, *ent-***3da**: CAS 848194-80-9, **3ea**: CAS 1001022-96-3, **3fa**: CAS 1187084-34-9, **3ga**: CAS 10001022-98-5.

### 3.3. Reduction of Nitro Group of ***3ea***

To a stirred solution of **3ea** (26.7 mg, 0.086 mmol) in MeOH (1.0 mL) were added NiCl_2_·6H_2_O (22.0 mg, 0.092 mmol) and NaBH_4_ (32. 5 mg, 0.86 mmol) at 0 °C and the resulting mixture was stirred at the same temperature for 1 h. The reaction was quenched with H_2_O, and the resulting biphasic mixture was extracted with ethyl acetate (x2). The combined organic layers were washed with brine and dried over Na_2_SO_4_. Volatiles were removed under reduced pressure and the resulting residue was purified by silica gel column chromatography (CHCl_3_/MeOH = 10/1) to give *tert -butyl(1S*,*2R)-2-amino-1-(4-methoxyphenyl)propylcarbamate* (**5ea**) as a colorless solid (23.9 mg, y. 99%). Colorless solid; M.p. 101–104 °C; IR (KBr) *ν* 1033, 1251, 1684, 3373; ^1^H-NMR (CDCl_3_) δ 1.00 (d, *J* = 6.4 Hz, 3H), 1.38 (s, 9H), 3.09–3.12 (m, 1H), 3.76 (s, 3H), 4.41 (brs, 1H), 5.39 (d, *J* = 7.4 Hz, 1H), 6.84 (d, *J* = 8.5 Hz, 2H), 7.15 (d, *J* = 8.5 Hz, 2H); ^13^C-NMR (CDCl_3_) δ 21.2, 28.3, 50.7, 55.2, 59.6, 79.2, 113.7, 127.3, 128.4, 155.4, 158.8; [α]_D_^26^ +41.0 (*c* 0.5, MeOH); ESI-MS *m*/*z* 303 [M+Na]^+^; HRMS (ESI-TOF) Anal. calcd. for C_15_H_24_N_2_NaO_3_ [M+Na]^+^*m*/*z* 303.1685, found 303.1682.

## 4. Conclusions

In summary, we developed a catalytic asymmetric nitro-Mannich reaction based on bifunctional heterobimetallic catalysis exerted by a rare earth metal/alkali metal heterobimetallic catalyst. The amide-based ligand **4a**, which was effective for the catalytic asymmetric nitroaldol reaction, proved to be a suitable platform for a Yb/K heterobimetallic catalyst in the asymmetric nitro-Mannich reaction, affording *anti*-1,2-nitroamines in up to 86% ee. Facile reduction of the nitro group of the product allowed for an efficient access to synthetically versatile enantioenriched 1,2-diamines.

## References

[1] Westermann B. (2003). Asymmetric catalytic aza-Henry reactions leading to 1,2-diamines and 1,2-diaminocarboxylic acids. Angew. Chem., Int. Ed..

[2] Marqués-López E., Merino P., Tejero T., Herrera R.P. (2009). Catalytic enantioselective aza-Henry reactions. Eur. J. Org. Chem..

[3] Ono N. (2001). The Nitro Group in Organic Synthesis.

[4] Ballini R., Petrini M. (2004). Recent synthetic developments in the nitro to carbonyl conversion (Nef reaction). Tetrahedron.

[5] Czekelius C., Carreira E.M. (2005). Convenient transformation of optically active nitroalkanes into chiral aldoximes and nitriles. Angew. Chem., Int. Ed..

[6] Yamada K.I., Harwood S.J., Gröger H., Shibasaki M. (1999). The first catalytic asymmetric nitro-Mannich-type reaction promoted by a new heterobimetallic complex. Angew. Chem., Int. Ed..

[7] Yamada K.-I., Moll G., Shibasaki M. (2001). The first enantioselective and diastereoselective catalytic nitro-Mannich reaction: a new entry to chiral vicinal diamines. Synlett.

[8] Nishiwaki N., Knudsen K.R., Gothelf K.V., Jørgensen K.A. (2001). Catalytic enantioselective addition of nitro compounds to imines - a simple approach for the synthesis of optically active α-nitro-β-Amino Esters. Angew. Chem. Int. Ed..

[9] Knudsen K.R., Risgaard T., Nishiwaki N., Gothelf K.V., Jørgensen K.A. (2001). The first catalytic asymmetric aza-Henry reaction of nitronates with imines: a novel approach to optically active β-nitro-α-amino acid- and α,β-diamino acid derivatives. J. Am. Chem. Soc..

[10] Lee A., Kim W., Lee J., Hyeon T., Kim B.M. (2004). Heterogeneous asymmetric nitro-Mannich reaction using a bis(oxazoline) ligand grafted on mesoporous silica. Tetrahedron: Asymmetry.

[11] Anderson J.C., Howell G.P., Lawrence R.M., Wilson C.S. (2005). An asymmetric nitro-Mannich reaction applicable to alkyl, aryl, and heterocyclic imines. J. Org. Chem..

[12] Trost B.M., Lupton D.W. (2007). Dinuclear zinc-catalyzed enantioselective aza-Henry reaction. Org. Lett..

[13] Handa S., Gnanadesikan V., Matsunaga S., Shibasaki M. (2007). *syn*-Selective catalytic asymmetric nitro-Mannich reactions using a heterobimetallic Cu−Sm−Schiff base complex. J. Am. Chem. Soc..

[14] Yoon T.P., Jacobsen E.N. (2005). Highly enantioselective thiourea-catalyzed nitro-Mannich reactions. Angew. Chem., Int. Ed..

[15] Xu X., Furukawa T., Okino T., Miyabe H., Takemoto Y. (2006). Bifunctional-thiourea-catalyzed diastereo- and enantioselective aza-Henry reaction. Chem. Eur. J..

[16] Bode C.M., Ting A., Schaus S.E. (2006). A general organic catalyst for asymmetric addition of stabilized nucleophiles to acyl imines. Tetrahedron.

[17] Robak M.T., Trincado M., Ellman J.A. (2007). Enantioselective aza-Henry reaction with an *N*-sulfinyl urea organocatalyst. J. Am. Chem. Soc..

[18] Wang C.J., Dong X.Q., Zhang Z.H., Xue Z.Y., Teng H.L. (2008). Highly *anti*-selective asymmetric nitro-Mannich reactions catalyzed by bifunctional amine-thiourea-bearing multiple hydrogen-bonding donors. J. Am. Chem. Soc..

[19] Rampalakos C., Wulff W.D. (2008). A novel bis-thiourea organocatalyst for the asymmetric aza-Henry reaction. Adv. Synth. Catal..

[20] Takada K., Nagasawa K. (2009). Enantioselective aza-Henry reaction with acyclic guanidine-thiourea bifunctional organocatalyst. Adv. Synth. Catal..

[21] Nugent B.M., Yoder R.A., Johnston J.N. (2004). Chiral proton catalysis: a catalytic enantioselective direct aza-Henry reaction. J. Am. Chem. Soc..

[22] Rueping M., Antonchick A.P. (2007). Brønsted-acid-catalyzed activation of nitroalkanes: a direct enantioselective aza-Henry reaction. Org. Lett..

[23] Palomo C., Oiarbide M., Laso A., López R. (2005). Catalytic enantioselective aza-Henry reaction with broad substrate scope. J. Am. Chem. Soc..

[24] Gomez-Bengoa E., Linden A., López R., Múgica- Mendiola I., Oiarbide M., Palomo C. (2008). Asymmetric aza-Henry reaction under phase transfer catalysis: an experimental and theoretical study. J. Am. Chem. Soc..

[25] Jiang X., Zhang Y., Wu L., Zhang G., Liu X., Zhang H., Fu D., Wang R. (2009). Doubly stereocontrolled asymmetric aza-Henry reaction with in situ generation of *N*-Boc-imines catalyzed by novel rosin-derived amine thiourea catalysts. Adv. Synth. Catal..

[26] Nitabaru T., Kumagai N., Shibasaki M. (2008). A catalytic asymmetric *anti*-selective nitroaldol reaction with a neodymium sodium heterobimetallic complex. Tetrahedron Lett..

[27] Nitabaru T., Nojiri A., Kobayashi M., Kumagai N., Shibasaki M. (2009). *anti*-Selective catalytic asymmetric nitroaldol reaction via a heterobimetallic heterogeneous catalyst. J. Am. Chem. Soc..

[28] Yamamoto H., Futatsugi K. (2005). “Designer acids”: combined acid catalysis for asymmetric synthesis. Angew. Chem.Int. Ed..

[29] Taylor M.S., Jacobsen E.N. (2006). Asymmetric catalysis by chiral hydrogen-bond donors. Angew. Chem.Int. Ed..

[30] Mukherjee S., Yang J.W., Hoffmann S., List B. (2007). Asymmetric enamine catalysis. Chem. Rev..

[31] Matsunaga S., Shibasaki M. (2008). Multimetallic bifunctional asymmetric catalysis based on proximity effect control. Bull. Chem. Soc. Jpn..

[32] Shibasaki M., Kanai M., Matsunaga S., Kumagai N. (2009). Recent progress in asymmetric bifunctional catalysis using multimetallic systems. Acc. Chem. Res..

[33] Mashiko T., Hara K., Tanaka D., Fujiwara Y., Kumagai N., Shibasaki M. (2007). En route to an efficient asymmetric synthesis of AS-3201. J. Am. Chem. Soc..

[34] Mashiko T., Kumagai N., Shibasaki M. (2008). An improved lanthanum catalytic system for asymmetric amination; toward a practical asymmetric synthesis of AS-3201 (ranirestat). Org. Lett..

[35] Nojiri A., Kumagai N., Shibasaki M. (2008). Asymmetric catalysis via dynamic substrate/ligand rare earth metal conglomerate. J. Am. Chem. Soc..

[36] Nojiri A., Kumagai N., Shibasaki M. (2009). Linking structural dynamics and functional diversity in asymmetric catalysis. J. Am. Chem. Soc..

[37] Mashiko T., Kumagai N., Shibasaki M. (2009). Managing highly coordinative substrates in asymmetric catalysis: a catalytic asymmetric amination with a lanthanum-based ternary catalyst. J. Am. Chem. Soc..

[38] Boc (1994). *tert*-butoxycarbonyl. *N*-Boc imines **1** were prepared by following the reported procedure; Kanazawa, A.M.; Denis, J.; Greene, A.E. Highly stereocontrolled and efficient preparation of the protected, esterification-ready docetaxel (taxotere) side chain. J. Org. Chem..

[39] Zhao X., Wang X.Z., Jiang X.K., Chen Y.Q., Li Z.T., Chen G.J. (2003). Hydrazide-based quadruply hydrogen-bonded heterodimers. Structure, assembling selectivity, and supramolecular substitution. J. Am. Chem. Soc..

[40] Li C., Ren S.F., Hou J.L., Yi H.P., Zhu S.Z., Jiang X.K., Li Z.T. (2005). F∙∙∙H–N hydrogen bonding driven foldamers: efficient receptors for dialkylammonium ions. Angew. Chem., Int. Ed..

[41] Reichardt C. (2003). Solvents and Solvent Effects in Organic Chemistry.

[42] The attempted reaction of **1a** and **2a** in the absence of ligand **4a** under otherwise identical conditions (Yb(O^i^Pr)_3_: 10 mol %, KHMDS: 20 mol %, THF, –60 °C, 22 h) afforded racemic **3aa** in 53% yield (determined by ^1^H-NMR, *anti/syn* = 2.6/1), strongly suggested that unidentified achiral basic species promoted the background reaction to decrease the stereoselectivity.

[43] The identical reaction in Table 5, entry 3 gave the product **3ca** after 20 h in 62% yield *anti/syn* = 18/1, 85% ee (*anti*), indicating the absence of retro-reaction and epimerization of the product during the reaction.

